# Supplementary material for: Glia-neuron interactions underlie state transitions to generalized seizures
Source: Nat Commun. 2019 Aug 23;10:3830. doi: 10.1038/s41467-019-11739-z (PMC6707163; doi:10.1038/s41467-019-11739-z)
Supplement: Supplementary file 1 — Supplementary Information [file 41467_2019_11739_MOESM1_ESM.pdf]

Supplementary Information

Glia-neuron interactions underlie state transitions to generalized seizures

Diaz Verdugo, Myren-Svelstad *et al*.

Supplementary Figures

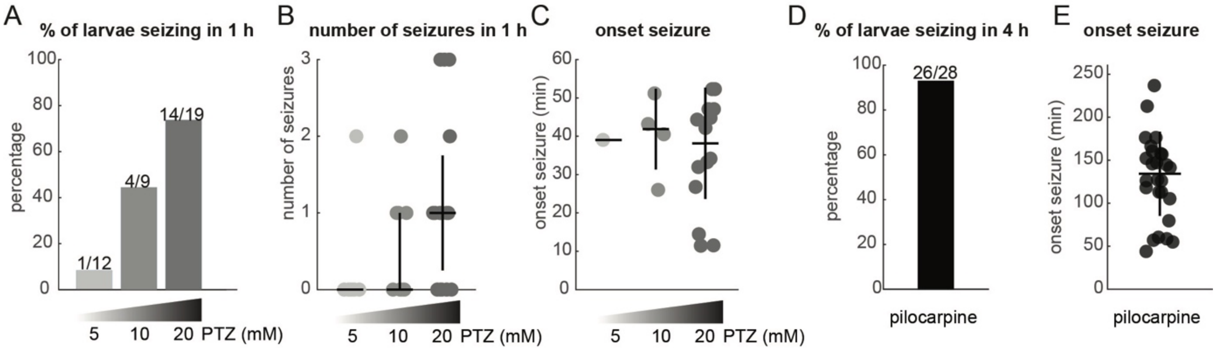

Supplementary Fig. 1. 20 mM pentylenetetrazole (PTZ) induces generalized seizures more efficiently than 5 mM and 10 mM PTZ, and faster than 60 mM pilocarpine

5 days old *Tg(elavl3:GCaMP6s)* and Tg(*GFAP:Gal4)nw7;Tg(UAS:GCaMP6s)* animals were imaged under an epifluorescence or two-photon microscope following treatment with 5 mM, 10 mM or 20 mM PTZ for 1 h (A-C) or 60 mM pilocarpine for 4 h (D-E). The percentage of animals presenting at least one generalized seizure during 1 h PTZ (A) or 4 h pilocarpine (D) treatment, the number of generalized seizures during 1 h PTZ treatment (B) and the onset of the first generalized seizure are quantified for each PTZ concentration (C) and for 60 mM pilocarpine (E). Error bars represent median and interquartile range. Numbers of observations are included on top of the bar graphs.

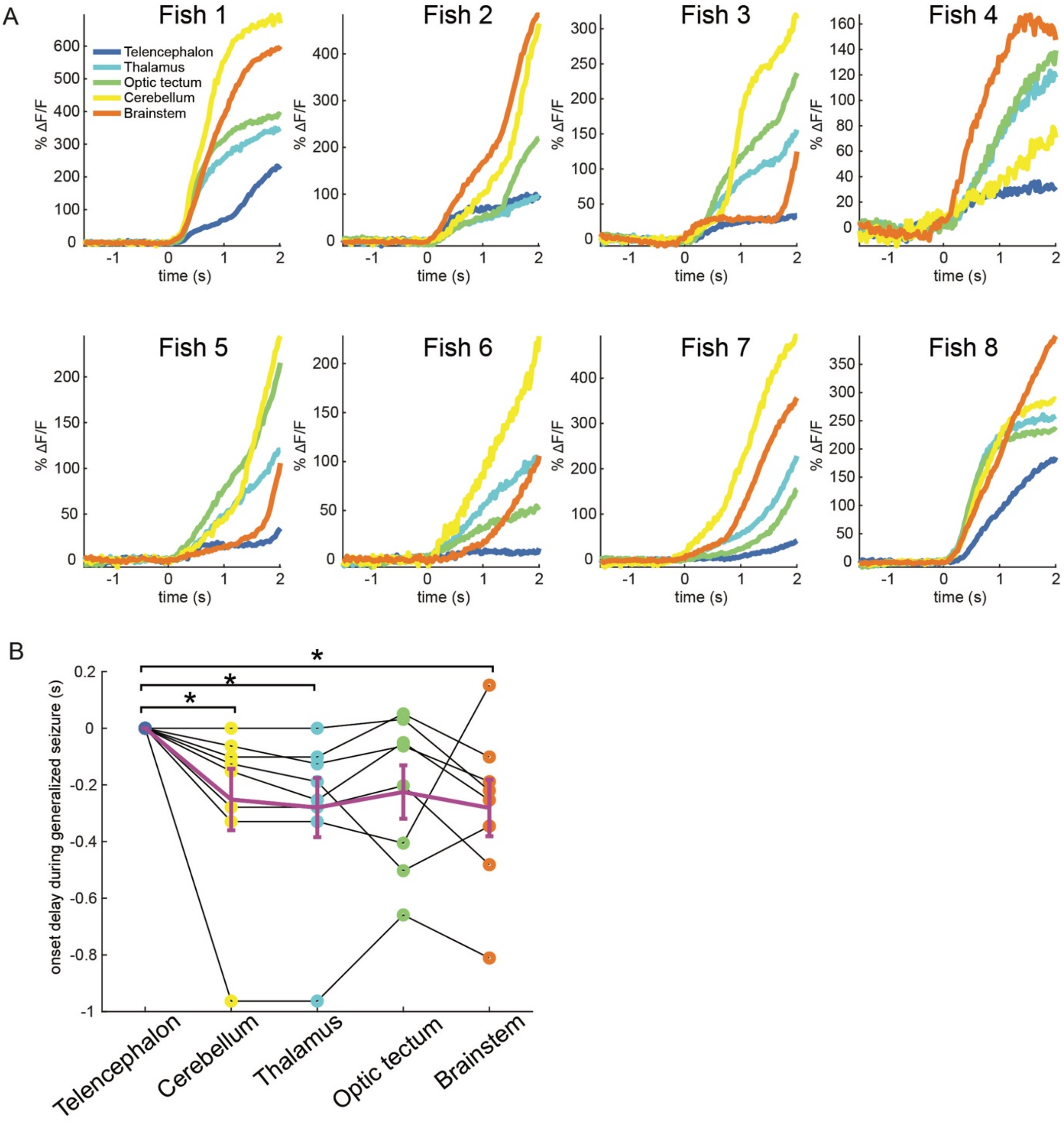

Supplementary Fig. 2. Telencephalon is recruited significantly later then other brain regions during generalized seizures.Related to Fig. 2-3

A) Average neural activity (ΔF/F) over time, organized by brain region in all recorded zebrafish with pentylenetetrazole (PTZ)-induced generalized seizures. The time period from 1 second before to 2 seconds after seizure onset is shown.

B) Telencephalon is recruited significantly later than most other brain regions (namely thalamus, cerebellum, and brainstem). Recruitment of telencephalon versus optic tectum does not show a significant difference.

*:$p<0.05$, Wilcoxon signed-rank test. Error bars (magenta) represent the s.e.m. of n=8 fish.

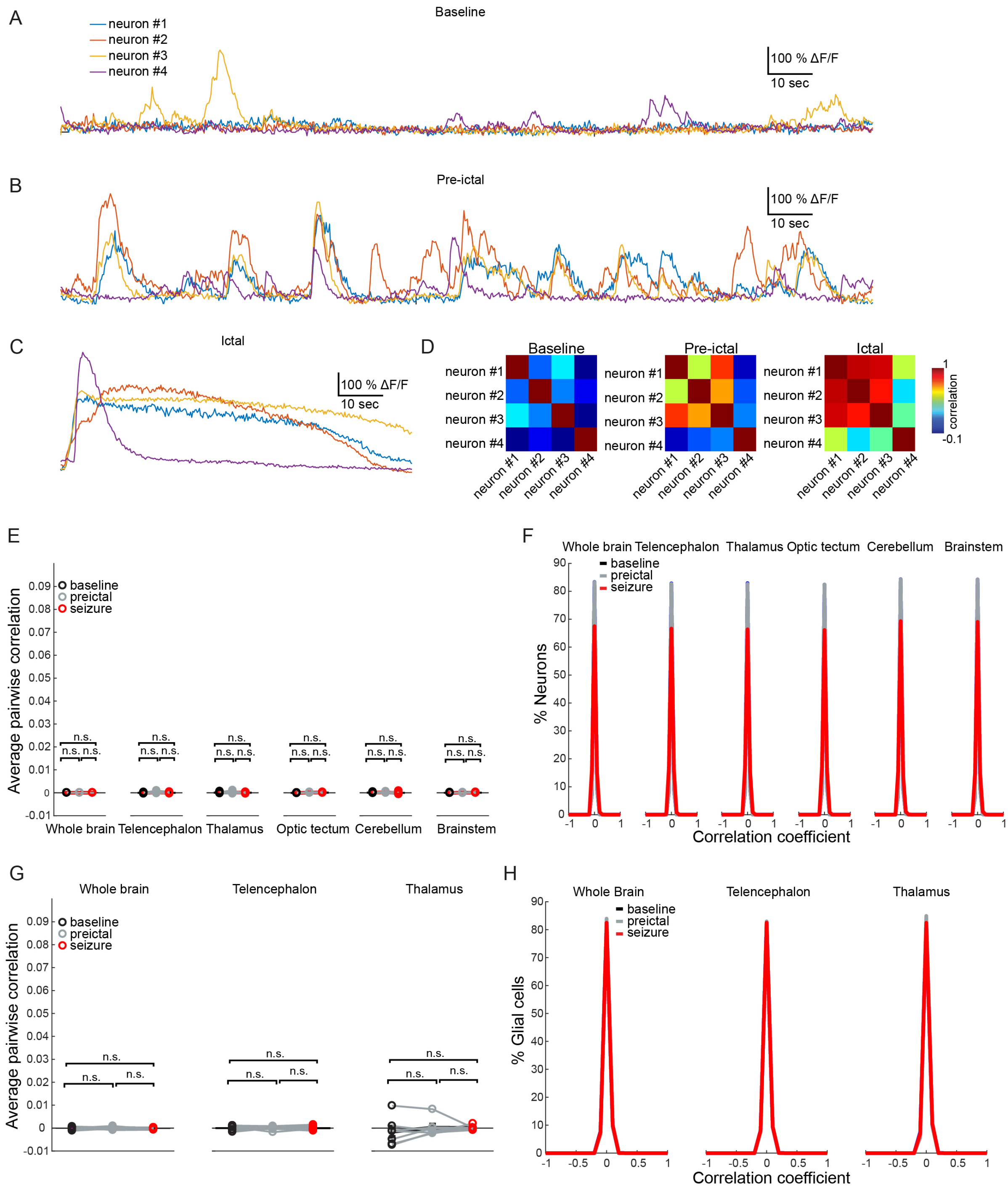

Supplementary Fig. 3. Single-neuron dynamics change significantly through state transitions, and correlations within neural and glial cell populations are not due to amplitude effects. Related to Fig. 2-4

A-C) Two photon calcium signals of four representative neurons during baseline, preictal, and seizure periods, in zebrafish larvae expressing *GCaMP6s* in all neurons, in response to 20 mM PTZ.

D) Correlations between pairs of neurons, which are presented in A-C. Note that neurons with highly synchronous activity (e.g. neurons 1 and 3) are highly correlated. Whereas neurons 2 and 4 exhibit asynchronous activity leading to low correlation coefficients.

E-F) Neural time series data (presented in Fig. 3B and 3E) were shuffled to show that neither average pairwise correlations nor correlation coefficients are due to amplitude effects.

G-H) Glial time series data (presented in Fig. 4G and 4I) were shuffled to show that neither average pairwise correlations nor correlation coefficients are due to amplitude effects.

n.s.= not significant ($p>0.05$), Wilcoxon signed-rank test.

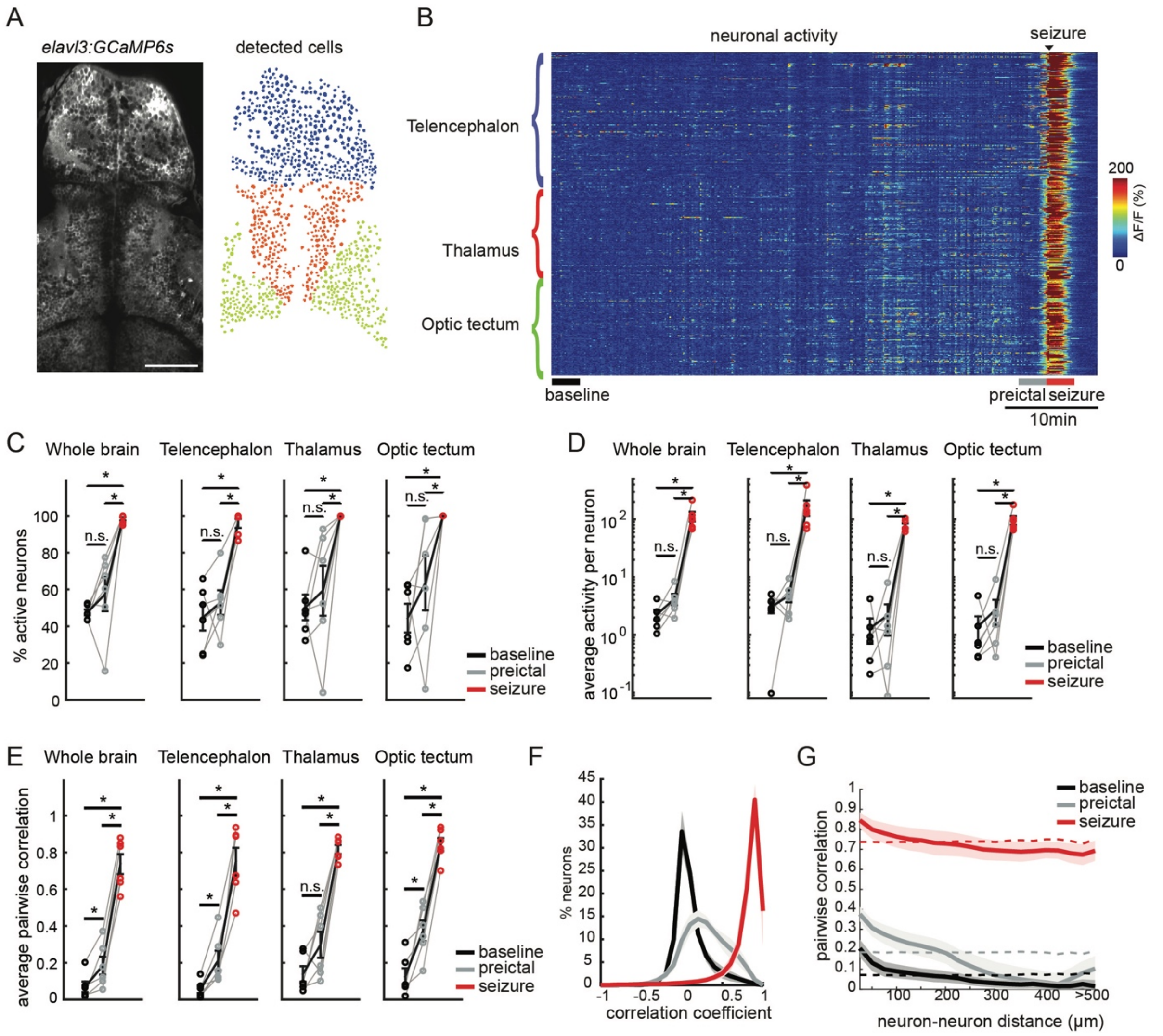

Supplementary Fig. 4. Pilocarpine-induced seizures exhibit features similar to pentylenetetrazole (PTZ)-induced seizures, but the ictogenesis occurs less rapidly

A) An optical section of a zebrafish larva expressing *GCaMP6s* in all neurons, obtained by two-photon microscopy, dorsal view (left). Individual neurons (right) in color-coded brain regions: telencephalon (blue), thalamus (red), and optic tectum (green). White bar reflects 50 μm.

B) Activity of individual neurons (ΔF/F) over time, organized by brain region. Application of 60 mM pilocarpine was done at the beginning of the recording. Baseline is indicated for a time period preceding strongly correlated activity. Warmer colors indicate stronger activity.

C) Percentage of active neurons ($>3std_{baseline}$) in the whole brain and per brain area during baseline (black), preictal (gray) and seizure (red) periods.

D) Average activity of the active neurons, defined by the area under the curve of the ΔF/F trace, in the whole brain and per brain area.

E) Average pairwise Pearson's correlation across the whole brain, and within individual brain regions.

F) Histogram representing the distribution of all correlation coefficients between neurons from all animals.

G) Relation between pairwise Pearson's correlation of neural activity and the distance between each neuron pair. Dotted lines represent the results when neuronal locations are shuffled. n = 6 fish. Shaded regions and error bars represent the s.e.m. (*p= <0.05, ns= not significant, Wilcoxon signed-rank test).

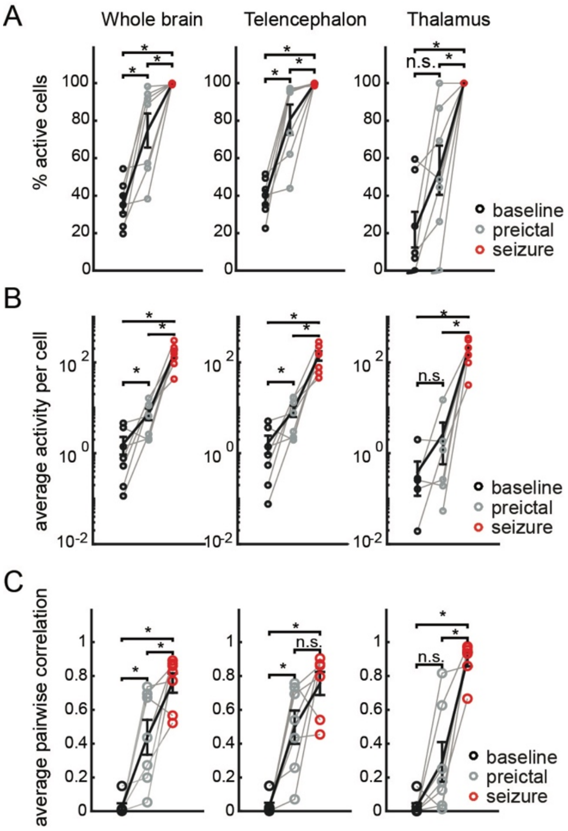

Supplementary Fig. 5. Glia from specific brain regions are differentially recruited during epileptic activity

Related to Fig. 4E-G. Percentage of active glial cells (>3$std_{baseline}$) during baseline (black), preictal (gray) and seizure (red) periods (A), average activity of the active glial cells, defined by the area under the curve of the ΔF/F trace (B) and average pairwise Pearson's correlation between glial cells (C) revealed that during the preictal period telencephalic glia are significantly more active and more correlated, when compared to baseline. This was not the case for thalamic glia (right). Each dot indicates one fish, n=7 fish. Error bars (black) represent the s.e.m.

(*p= <0.05, ns= not significant Wilcoxon signed-rank test).

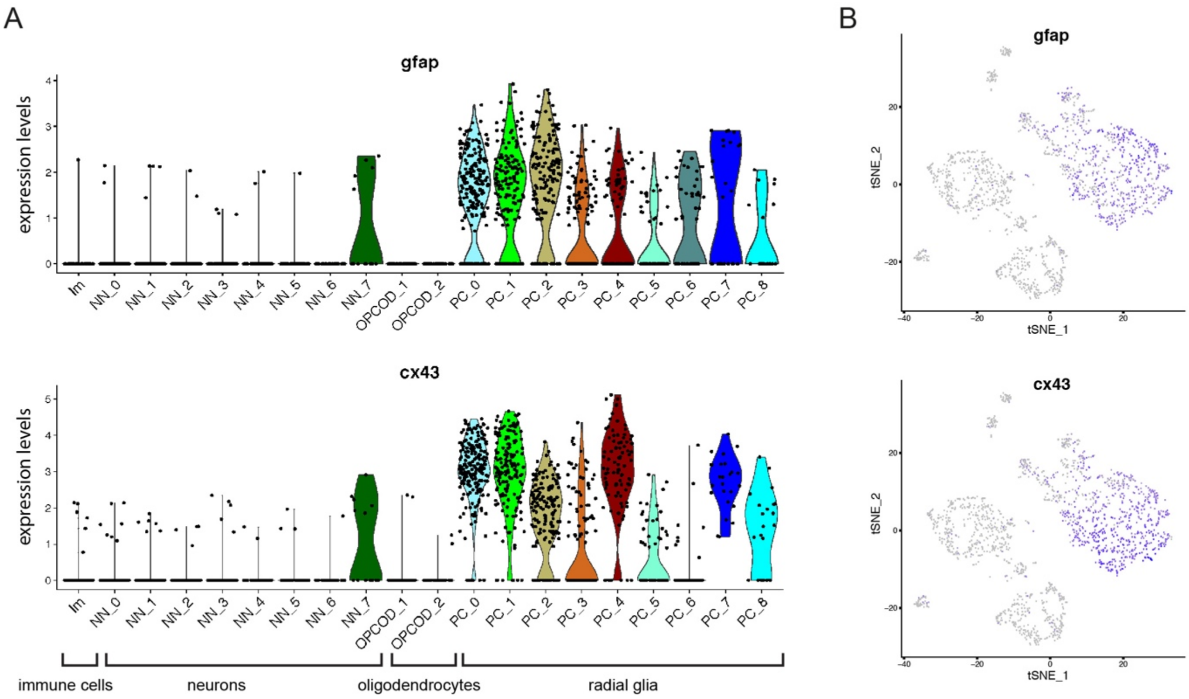

Supplementary Fig. 6. Connexin 43 is highly expressed in zebrafish radial glial cells

Single cell transcriptomics data of adult zebrafish telencephalon deposited in https://kizillab.org/singlecell[1] reveal a strong overlap between *GFAP* expression marking glial cells and *connexin 43* in expression (A) and tSNE plots highlighting the overlap of *GFAP* expressing and *connexin 43* expressing cell clusters (B).

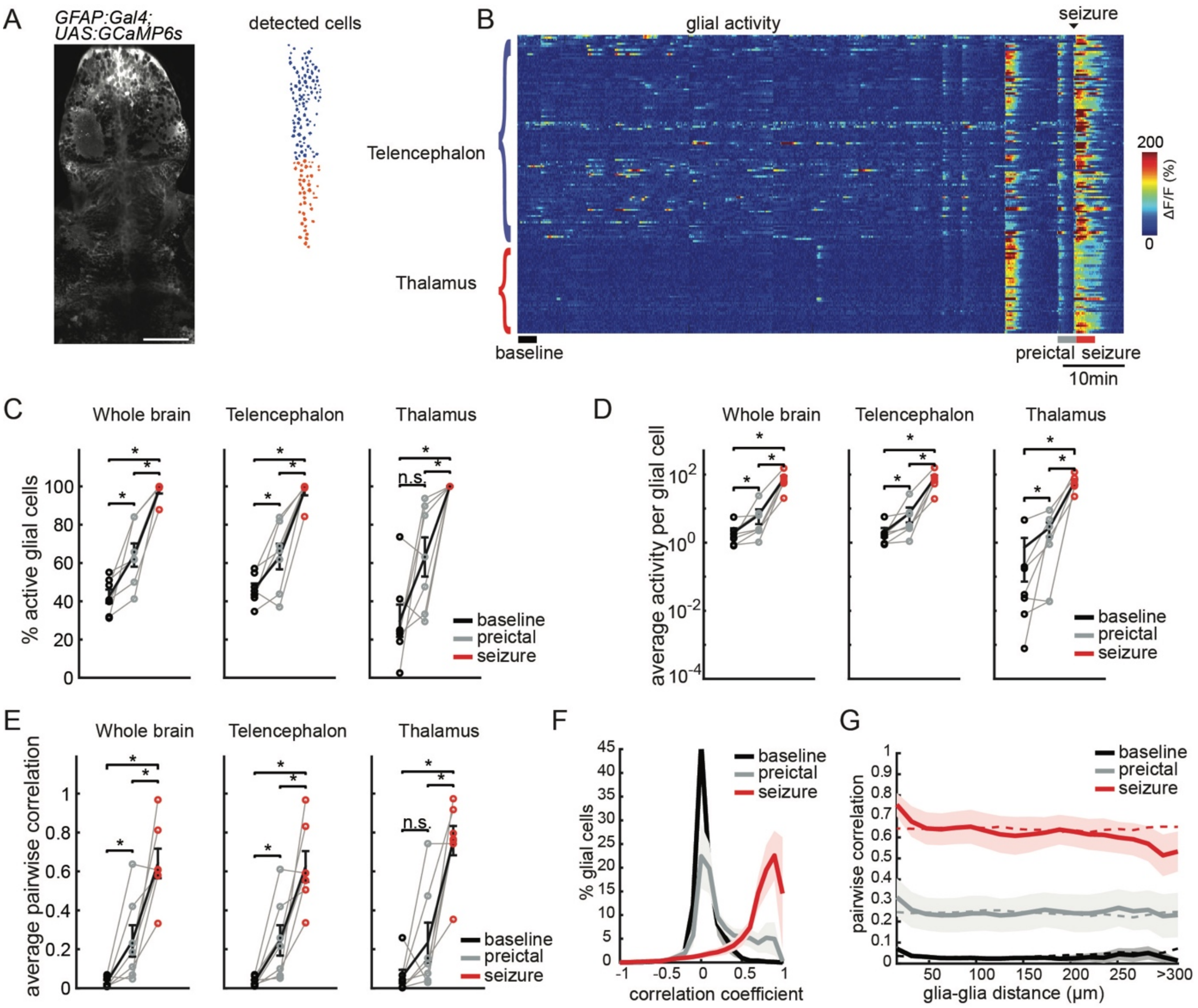

Supplementary Fig. 7. Pilocarpine-induced seizures are preceded by strong glial activity and synchrony, similar to pentylenetetrazole (PTZ)-induced seizures

A) An optical section of a zebrafish larva expressing *GCaMP6s* in *GFAP* positive radial glia, obtained by two-photon microscopy, dorsal view (left). Individual glial cells (right) in color-coded brain regions: telencephalon (blue), and thalamus (red). White bar reflects 50 μm.

B) Activity of individual glial cells (ΔF/F) over time, organized by brain region. Application of 60 mM pilocarpine was done at the beginning of the recording. Baseline is indicated for a time period preceding strongly correlated activity. Warmer colors indicate stronger activity.

C) Percentage of active glial cells (>3std$_{baseline}$) in the whole brain and per brain area during baseline (black), preictal (gray) and seizure (red) periods.

D) Average activity of the active glial cells, defined by the area under the curve of the ΔF/F trace, in the whole brain and per brain area.

E) Average pairwise Pearson's correlation across the whole brain, and within individual brain regions.

F) Histogram representing the distribution of all correlation coefficients between glial cells from all animals.

G) Relation between pairwise Pearson's correlation of glial activity and the distance between each glia pair. Dotted lines represent the results when glial locations are shuffled.

n=7 fish. Shaded regions and error bars represent the s.e.m. (*p= <0.05, ns= not significant, Wilcoxon signed-rank test).

Supplementary Tables

Supplementary Table 1. Plasmids and zebrafish lines used in this study

Reagent or Resource	Source	Identifier
**Plasmid DNA**		
GFAP:Gal4	Ohshima lab	[2]
tol2 transposase pCS2FA plasmid	http://tol2kit.genetics.utah.edu/index.php/Main_Page	[3]
**Zebrafish lines**		
Tg(elavl3:GCaMP6s)	[4]	ZFIN Cat# ZDB-ALT-141023-1, RRID:ZFIN_ZDB-ALT-141023-1
Tg(GFAP:Gal4)nw7	This study	
Tg(5xUAS-hsp70l:GCaMP6s)	[5]	ZFIN Cat# ZDB-ALT-170615-4
Tg(UAS:Cr.ChR2_H134R-mCherry)	[6,7]	ZFIN Cat# ZDB-ALT-101227-5, RRID:ZFIN_ZDB-ALT-101227-5
Et(-0.6hsp70l:Gal4-VP16)s1020t	[8]	ZFIN Cat# ZDB-ALT-070420-21, RRID:ZFIN_ZDB-ALT-070420-21
Tg(elavl3:jRCaMP1a)	[9]	ZFIN Cat# ZDB-ALT-160519-3, RRID:ZFIN_ZDB-ALT-160519-3
Tg(gad1:GFP)	[10]	ZFIN Cat# ZDB-ALT-131127-6, RRID:ZFIN_ZDB-ALT-131127-6
Tg(vglut2a:dsRed)	[11]	ZFIN Cat# ZDB-ALT-100505-2, RRID:ZFIN_ZDB-ALT-100505-2
Tg(gfap:Eco.GltL-cpEGFP)	[12]	ZFIN Cat# ZDB-ALT-170404-12

Supplementary Table 2. Chemicals, software, algorithms, and other resources used in this study

Reagent or Resource	Source	Identifier
**Chemicals**		
Pentylenetetrazole (PTZ)	Sigma-Aldrich	Cat# P6500
MS222 (Tricaine methanesulfonate)	Sigma-Aldrich	Cat# E10521
Pilocarpine	Tocris	Cat # 0694
LMP Agarose	Fisher Scientific	Cat# 16520100
α-bungarotoxin	Thermo Fisher Scientific	Cat# B1601
Neurobiotin	Vector labs	Cat# SP-1125
PBS	Thermo Fisher Scientific	Cat# BR0014G
Streptavidin	Vector labs	Cat# SA-5000
**Software and Algorithms**		
ImageJ/Fiji	https://fiji.sc	
Cell detection	[13]	
Image alignment and processing	[14]	
**Other**		
Pressure injector	Eppendorf	Femtojet 4i
Confocal microscope (20x plan NA 0.8 objective)	Zeiss	Examiner Z1
Stereomicroscope (20x Plan-Apochromat, NA 0.8)	Zeiss	Axio Imager M1
Two-photon microscope	Thorlabs	
Two-photon microscope	Scientifica	
Epifluorescence microscope	Olympus Corporation	Olympus BX51 fluorescence microscope
MultiClamp 700B amplifier, Digidata	Axon instruments	
1440A digitizer	Axon instruments	
EMCCD camera	Hamamatsu Photonics	
Sutter Laser puller	Sutter	Model P-200

Supplementary References

1 Cosacak, M. I. *et al.* Single-Cell Transcriptomics Analyses of Neural Stem Cell Heterogeneity and Contextual Plasticity in a Zebrafish Brain Model of Amyloid Toxicity. *Cell Rep* **27**, 1307-1318.e1303, doi:10.1016/j.celrep.2019.03.090 (2019).

2 Shimizu, Y., Ito, Y., Tanaka, H. & Ohshima, T. Radial glial cell-specific ablation in the adult Zebrafish brain. *Genesis* **53**, 431-439, doi:10.1002/dvg.22865 (2015).

3 Kwan, K. M. *et al.* The Tol2kit: a multisite gateway-based construction kit for Tol2 transposon transgenesis constructs. *Dev Dyn* **236**, 3088-3099, doi:10.1002/dvdy.21343 (2007).

4 Vladimirov, N. *et al.* Light-sheet functional imaging in fictively behaving zebrafish. *Nature methods* **11**, 883-884, doi:10.1038/nmeth.3040 (2014).

5 Muto, A. *et al.* Activation of the hypothalamic feeding centre upon visual prey detection. *Nat Commun* **8**, 15029, doi:10.1038/ncomms15029 (2017).

6 Hubbard, Jeffrey M. *et al.* Intraspinal Sensory Neurons Provide Powerful Inhibition to Motor Circuits Ensuring Postural Control during Locomotion. *Current Biology* **26**, 2841-2853, doi:10.1016/j.cub.2016.08.026 (2016).

7 Schoonheim, P. J., Arrenberg, A. B., Del Bene, F. & Baier, H. Optogenetic localization and genetic perturbation of saccade-generating neurons in zebrafish. *J Neurosci* **30**, 7111-7120, doi:10.1523/JNEUROSCI.5193-09.2010 (2010).

8 Scott, E. K. & Baier, H. The cellular architecture of the larval zebrafish tectum, as revealed by gal4 enhancer trap lines. *Frontiers in neural circuits* **3**, 13-13, doi:10.3389/neuro.04.013.2009 (2009).

9 Dunn, T. W. *et al.* Brain-wide mapping of neural activity controlling zebrafish exploratory locomotion. *Elife* **5**, e12741, doi:10.7554/eLife.12741 (2016).

10 Satou, C. *et al.* Transgenic tools to characterize neuronal properties of discrete populations of zebrafish neurons. *Development* **140**, 3927-3931, doi:10.1242/dev.099531 (2013).

11 Miyasaka, N. *et al.* From the olfactory bulb to higher brain centers: genetic visualization of secondary olfactory pathways in zebrafish. *J Neurosci* **29**, 4756-4767, doi:10.1523/JNEUROSCI.0118-09.2009 (2009).

12 MacDonald, R. B., Kashikar, N. D., Lagnado, L. & Harris, W. A. A Novel Tool to Measure Extracellular Glutamate in the Zebrafish Nervous System In Vivo. *Zebrafish* **14**, 284-286, doi:10.1089/zeb.2016.1385 (2017).

13 Ohki, K., Chung, S., Ch'ng, Y. H., Kara, P. & Reid, R. C. Functional imaging with cellular resolution reveals precise micro-architecture in visual cortex. *Nature* **433**, 597, doi:10.1038/nature03274 (2005).

14 Reiten, I. *et al.* Motile-Cilia-Mediated Flow Improves Sensitivity and Temporal Resolution of Olfactory Computations. *Current Biology* **27**, 166-174, doi:10.1016/j.cub.2016.11.036 (2017).
