# Supplementary material for: Glia-neuron interactions underlie state transitions to generalized seizures
Source: Nat Commun. 2019 Aug 23;10:3830. doi: 10.1038/s41467-019-11739-z (PMC6707163; doi:10.1038/s41467-019-11739-z)
Supplement: Supplementary file 4 — Description of Additional Supplementary Files [file 41467_2019_11739_MOESM4_ESM.pdf]

Description of Additional Supplementary Files

File Name: Supplementary Movie 1
Description: Two-photon calcium imaging of Tg(elavl3:GCaMP6s) transgenic zebrafish larva expressing GCaMP6s in all neurons, perfused with 20 mM pentylenetetrazole (PTZ).

File Name: Supplementary Movie 2
Description: Two-photon recording of Tg(GFAP:Gal4)nw7; Tg(UAS:GCaMP6s) transgenic zebrafish larva expressing GCaMP6s in all radial glia, perfused with 20 mM pentylenetetrazole (PTZ).

File Name: Supplementary Movie 3
Description: Two-photon calcium imaging of Tg(elavl3:GCaMP6s) transgenic zebrafish larva expressing GCaMP6s in all neurons, upon application of 60 mM pilocarpine.

File Name: Supplementary Movie 4
Description: Two-photon recording of Tg(GFAP:Gal4)nw7; Tg(UAS:GCaMP6s) transgenic zebrafish larva expressing GCaMP6s in all radial glia, upon application of 60 mM pilocarpine.
